# “Wanna cry this out real quick?”: an examination of secondary traumatic stress risk and resilience among post-overdose outreach staff in Massachusetts

**DOI:** 10.1186/s12954-024-00975-2

**Published:** 2024-03-19

**Authors:** Samantha F. Schoenberger, Emily R. Cummins, Jennifer J. Carroll, Shapei Yan, Audrey Lambert, Sarah M. Bagley, Ziming Xuan, Traci C. Green, Franklin Cook, Amy M. Yule, Alexander Y. Walley, Scott W. Formica

**Affiliations:** 1 Boston Medical Center, Grayken Center for Addiction, Clinical Addiction Research and Education Unit, Section of General Internal Medicine, Department of Medicine, Boston University School of Medicine, 801 Massachusetts Ave, 2nd Floor, Boston, MA 02118 USA; 2 Department of Sociology & Anthropology, North Carolina State University, 10 Current Drive, Raleigh, NC 27605 USA; 3 Department of Medicine, Brown University, 222, Richmond St. Providence, 02903 USA; 4 Access, Harm Reduction, Overdose Prevention and Education (AHOPE), Boston Public Health Commission, 774 Albany Street, Access, Boston, MA 02118 USA; 5 Division of General Pediatrics, Department of Pediatrics, Boston University School of Medicine, Boston Medical Center, Boston, MA 02118 USA; 6 Department of Community Health Sciences, Boston University School of Public Health, 801 Massachusetts Avenue, Boston, MA 02118 USA; 7 The Heller School for Social Policy and Management, Brandeis University, 415 South Street, Waltham, MA 02453 USA; 8 Peer Support Community Partners, 30 Brimmer Street, Watertown, MA 02472 USA; 9 Department of Psychiatry, Boston Medical Center, Boston University School of Medicine, Boston, MA 02118 USA; 10 Social Science Research and Evaluation, Inc, 84 Mill Street, Lincoln, MA 01773 USA

**Keywords:** Post-overdose outreach, Overdose, Public health, Occupational stress, Compassion fatigue

## Abstract

**Background:**

Post-overdose outreach programs engage overdose survivors and their families soon after an overdose event. Staff implementing these programs are routinely exposed to others’ trauma, which makes them vulnerable to secondary traumatic stress (STS) and compassion fatigue. The purpose of this study was to explore experiences of STS and associated upstream and downstream risk and protective factors among program staff.

**Methods:**

We conducted a post-hoc analysis of semi-structured interviews with post-overdose outreach program staff in Massachusetts. Transcripts were analyzed using a multi-step hybrid inductive-deductive approach to explore approaches and responses to outreach work, factors that might give rise to STS, and compassion fatigue resilience. Findings were organized according to the three main constructs within Ludick and Figley’s compassion fatigue resilience model (empathy, secondary traumatic stress, and compassion fatigue resilience).

**Results:**

Thirty-eight interviews were conducted with staff from 11 post-overdose outreach programs in Massachusetts. Within the empathy construct, concern for others’ well-being emerged as a motivator to engage in post-overdose outreach work – with staff trying to understand others’ perspectives and using this connection to deliver respectful and compassionate services. Within the secondary traumatic stress construct, interviewees described regular and repeated exposure to others’ trauma – made more difficult when exposures overlapped with staff members’ personal social spheres. Within the compassion fatigue resilience construct, interviewees described the presence and absence of self-care practices and routines, social supports, and workplace supports. Job satisfaction and emotional detachment from work experiences also arose as potential protective factors. Interviewees reported inconsistent presence and utilization of formal support for STS and compassion fatigue within their post-overdose outreach teams.

**Conclusion:**

Post-overdose outreach program staff may experience secondary traumatic stress and may develop compassion fatigue, particularly in the absence of resilience and coping strategies and support. Compassion fatigue resilience approaches for post-overdose outreach staff warrant further development and study.

## Introduction

Post-overdose outreach programs, which engage overdose survivors and/or their social networks in the days after an overdose event, are an emerging response to opioid overdose [1–5]. Many such programs in the Commonwealth of Massachusetts are collaborations between public safety (i.e., police officers or fire fighters) and public health professionals (i.e., behavioral health personnel, recovery coaches, peer workers, or harm reduction outreach workers) [4]. In 2019, post-overdose outreach programs that conduct home-based outreach were operating in 44% (156/351) of municipalities in Massachusetts [4]. These programs provide a range of services including referral to and navigation of addiction treatment systems, recovery support, overdose prevention education and naloxone distribution, and support for families and social networks of overdose survivors [5]. Implementation of these programs has been associated with lower rates of fatal opioid overdose at the municipal level [6], but the operational pathways through which they operate is not well understood [7] – including critical questions concerning whether incorporating law enforcement officers into outreach activities increases overdose survivors’ risk of arrest and other adverse outcomes [8–12]. Independent of team composition and team members’ background and training, post-overdose outreach work can cause mental stress among team members due to their close interactions with overdose survivors and families who have recently experienced a life-threatening event [13–15].

In 1992, Judith Herman, a prominent psychiatrist, wrote “trauma is contagious” (p. 140) to describe the phenomenon of *traumatic countertransference* or *vicarious traumatization*, the process by which therapists treating patients for symptoms associated with post-traumatic stress disorder become, themselves, at risk of adverse psychological and physical health consequences via that therapeutic work [16]. Though Herman was strictly referring to this narrowly defined segment of physician-patient interaction, this concept has since diffused across disciplines, has been applied to different workforces (e.g., medical providers, social workers, emergency first responders), and has been interchangeably referred to as *secondary traumatic stress*, *compassion fatigue*, and *professional burnout* [17–21]. In 1995, Figley [17] attempted to unify these overlapping concepts and terms, arguing that they collectively cover what he described as, “the natural and consequential behaviors and emotions resulting from knowing about a traumatizing event experienced by a significant other and the stress resulting from helping or wanting to help a traumatized or suffering person” (p.7). In contrast, Newell and MacNeil [22] note these terms are distinct though often erroneously interchanged. They draw subtle distinctions between: *vicarious traumatization* (changes in internal cognition due to exposure to others’ trauma); *secondary traumatic stress* (external behavioral symptoms); *professional burnout* (mental and physical exhaustion based on chronic exposure to stressful situations); and *compassion fatigue* (a combination of symptoms of secondary traumatic stress and professional burnout) [22]. Though professional debates about the unity or distinctiveness of these conditions continue, scholars have argued that these conditions (even if variably labelled and defined) are manifest and observable through a range of physical, mental, and behavioral symptoms—what Mark Nichter referred to as “idioms of distress” [23]—such as sleeplessness, headaches, fatigue, workplace absenteeism, job turn-over, emotional distress, anxiety, depression, and symptoms paralleling posttraumatic stress disorder [17, 21, 24].

Recent events in the social, political, and public health landscapes have resulted in increased scholarly focus on this domain. This has included examinations of Secondary Traumatic Stress (STS) and compassion fatigue among healthcare workers, emergency first responders, and educators during the COVID-19 pandemic [25–27]; police officers in response to the public tensions over racially inequitable policing practices [28]; and first responders, harm reductionists, and peer workers within the context of drug overdose prevention, management, and response [29–33]. Regarding the latter, Winstanley [29] proposed the existence of *overdose-related compassion fatigue* to describe communal and personal distress resulting from knowledge of or exposure to overdose and overdose reversal events. Related lines of inquiry have examined the presence of STS and compassion fatigue among emergency first responders dispatched to respond to overdose events [34–36] and explored the common stresses and occupational hazards that characterize work in overdose prevention sites and other harm reduction settings [30, 31, 37–39].

An individual’s propensity to experience STS and compassion fatigue is hypothesized to be related to upstream and downstream risk and protective factors that exacerbate or insulate workers from harm [40]. In their compassion fatigue resilience model, Ludick and Figley [41] describe how health/mental health workers and first responders experience ebbs and flows of STS and explore why some are more susceptible and others are less susceptible to this reaction. On the upstream side, they view an individual’s empathic ability, the “capability and proclivity to recognize suffering in others” [41, p.117] as essential for building rapport and providing effective front-line services; however, this ability also produces vulnerability to STS and compassion fatigue – particularly in the absence of effective coping strategies and meaningful professional or psychological support [41]. Previous qualitative research suggests that the presence of such supports vary across post-overdose outreach programs, with some offering no professional or psychological supports to outreach team members at all [9]. Previous data from our research group indicated that just over half (53%) of post-overdose outreach programs surveyed in Massachusetts reported establishing protocols for supporting post-overdose outreach team members affected by grief [4]. On the downstream side, Ludick and Figley posit risk to be elevated when individuals experience prolonged exposure to stressors and compartmentalize stress reactions, but lessened when individuals establish self-care routines, disengage when not working, have a sense of job satisfaction, and perceive adequate social support [41].

To the best of our knowledge, no studies have explored the conditions that might give rise to STS and compassion fatigue among multidisciplinary teams formed for post-overdose outreach or the need for interventions to mitigate their impact. Therefore, existing support for post-overdose outreach workers may be insufficient. Overdose responders in previous studies reported a lack of necessary occupational resources, such as paid leave, and work and life boundaries with which to cope with the mental toll of service work [42–44]. Within the context of post-overdose outreach programs, failure to proactively assess and address STS and compassion fatigue has the potential to contribute to personal and professional burnout among outreach team members. This could, in turn, compromise the expressed goals of outreach, re-traumatize overdose survivors and their social networks, and result in decreased levels of engagement, quality of interactions during outreach, and ultimately, result in worse health outcomes for overdose survivors. The purpose of this study was to explore potential markers that might contribute to or mitigate the experience of STS and compassion fatigue among post-overdose outreach team members. We examined interview data from post-overdose outreach programs in Massachusetts using Ludick and Figley’s [41] compassion fatigue resilience model as a framework for organizing and interpreting the findings.

## Methods

### Study design and setting

This post-hoc study analyzed interview data collected as part of the qualitative component of a cross-sectional, mixed method explanatory sequential examination [9] of post-overdose outreach programs in Massachusetts (United States) conducted between September 2018 and March 2022. In summary, the larger study identified 157 post-overdose outreach programs that were operating in Massachusetts prior to July 2019. A detailed survey completed by 138 of these programs was used to characterize their components (e.g., team composition, outreach approach, services provided) [4] and to explore associations between the presence of these programs and subsequent reductions in opioid overdose deaths [6]. The qualitative aim of the larger study involved conducting semi-structured interviews with 38 post-overdose outreach team members at 11 of these programs to further explore patterns observed in the quantitative data related to the implementation of these programs [8, 9]. Primary data for the present study were drawn from questions related to potentially stressful exposures during outreach and to how teams and team members process and cope with these exposures.

### Theoretical framework

In this study, we were interested in understanding whether post overdose outreach workers demonstrated markers of the collection of constructs variably referred to as STS, compassion fatigue, and professional burnout. We were also interested in exploring whether programs or workers themselves were taking any steps to mitigate and minimize the impacts of working conditions that predispose them to this constellation of impacts. We found Ludick and Figley’s compassion fatigue resilience model [41] to be the best fit with our data given the diverse professional backgrounds and experiences represented in our study sample and the model’s focus on upstream and downstream risk and protective factors as well as its acknowledgement that individuals do not uniformly experience or cope with STS and compassion fatigue in the same way. This framework was identified post-hoc following data collection and was not used to inform the interview protocol described below.

**Fig. 1:**
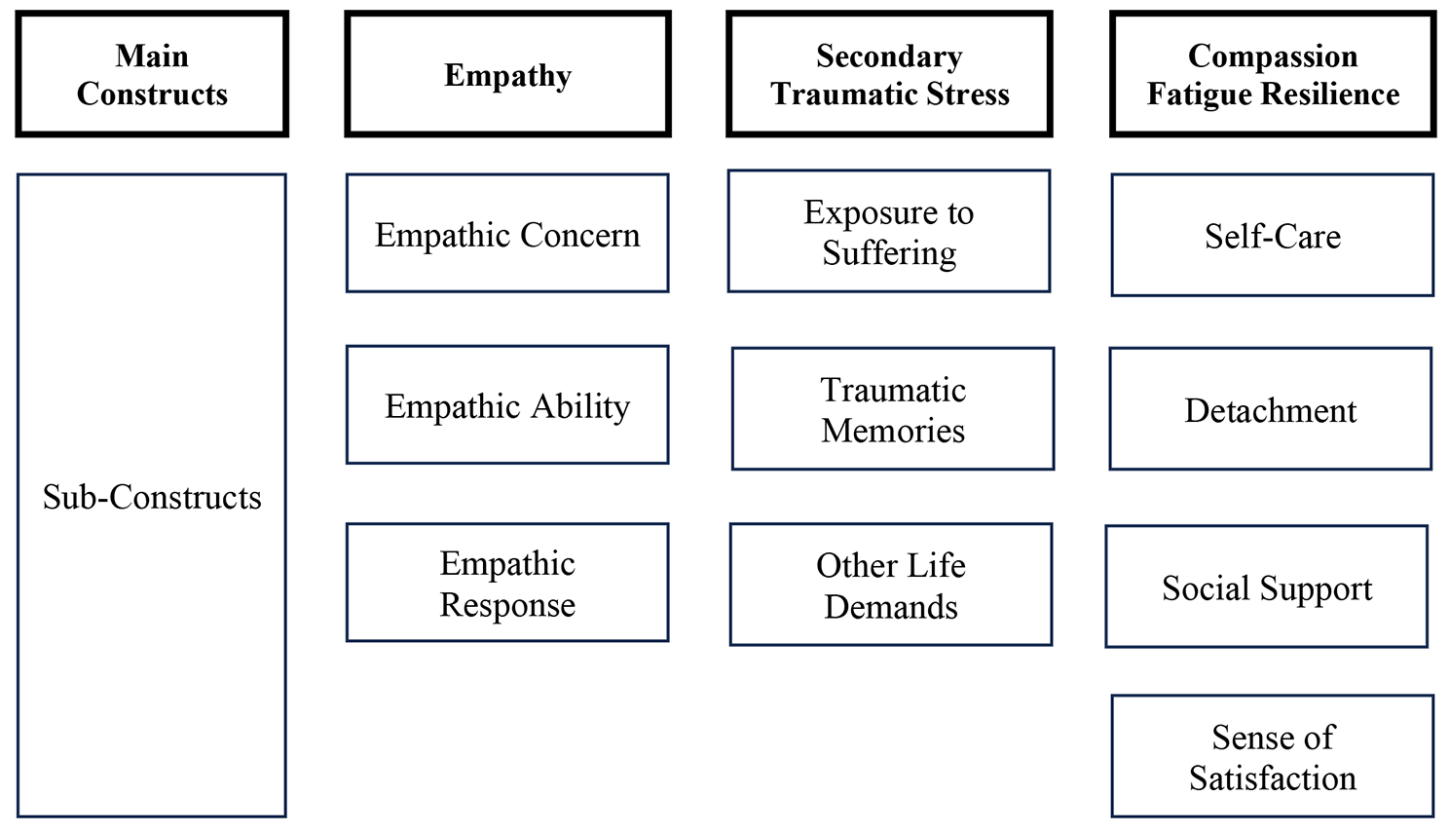
Main constructs and sub-constructs in compassion fatigue resilience model [41]

Ludick and Figley’s compassion fatigue resilience model (see Fig. 1) uses the terms secondary traumatic stress and compassion fatigue synonymously, noting “compassion fatigue is the term favored for helping professions whereas STS is used across diverse populations” [41, p.112]. For the remainder of this paper, we use the terms STS and compassion fatigue together to represent the continuum of exposure to others’ trauma and resulting impacts on a workforce members’ mental, emotional, and physical health.

We center the three main constructs from the compassion fatigue resilience model [41] in our analysis: empathy, secondary traumatic stress, and compassion fatigue resilience, and explore several of the model’s sub-constructs within each area. Within the compassion fatigue resilience model, empathy is identified as an upstream factor that motivates people to work in helping professions and makes them effective at their jobs, but also makes them susceptible to STS and compassion fatigue – colloquially referred to as “the cost of caring.” The empathy construct is sub-divided into three component parts – an individual’s compassion and interest in helping others (*empathic concern*), their capacity to recognize suffering in others (*empathic ability*), and their efforts to reduce the suffering of others (*empathic response*). In the context of post-overdose outreach, this might be represented by an outreach worker expressing concern for those encountered during outreach, empathizing with or adopting the perspective of outreach recipients, and delivering services in a compassionate and respectful manner.

In Ludick and Figley’s model [41], the terms STS and compassion fatigue represent the manifestation of physical, mental, and behavioral health issues that can arise from regular exposure to the suffering of others (*prolonged exposure to suffering*). They also hypothesize susceptibility to STS and compassion fatigue to be influenced by *personal trauma history* and *other life demands* (e.g., financial difficulties, familial stresses, personal history with incarceration or substance use disorder), but these latter factors were beyond the scope of the interview protocol in this study. In this study, we primarily explored how experiences and consequences of prolonged exposure to suffering might be evident in the details of firsthand or secondhand accounts of events encountered while conducting post-overdose outreach.

The final construct within the compassion fatigue resilience model includes downstream adaptation, coping strategies, and support hypothesized to protect and insulate individuals from STS and compassion fatigue. These strategies and related factors include having established *self-care* routines, *detachment* from work during non-working hours, having a high sense of *job satisfaction*, and the perception of adequate *social support*. For post-overdose outreach workers, this might include accounts of exercising, respecting a healthy work-life balance, deriving pleasure and a sense of fulfillment from work, and having robust personal and professional networks and supportive relationships.

### Data collection and measures

Semi-structured interviews were conducted (by EC, a qualitative sociologist) with 38 post-overdose outreach staff between December 2019 and August 2020. Eligible interviewees were individuals who were at least 18 years of age at the time of the interview, a member of one of the 138 post-overdose outreach programs that completed the detailed program survey, and actively engaged in post-overdose outreach work within the 12 months preceding the interview. A purposive sampling strategy was used to elicit a broad range of experiences and perspectives based on factors such as professional background (e.g., police officers, recovery coaches, harm reductionists, fire fighters, substance use disorder counselors), geographic location within the Commonwealth, community demographics, and program structure. Interviews were conducted in-person between December 2019 and February 2020 and then virtually from March 2020 to August 2020 as necessitated by COVID-19 physical distancing restrictions.

The full semi-structured interview guide included *a priori* domains focusing on descriptions of program goals, different outreach approaches adopted by programs, team member experiences during outreach visits, perceived efficacy of services, and challenges encountered. *A priori* domains relevant to the current study included post-visit debriefing protocols (“How does the team debrief after a visit?”), how team members dealt with exposure to the trauma of others (“Does your team do anything to deal with trauma that you might experience as outreach workers?”), and detailed descriptions of post-overdose outreach visits. Participants were compensated with a $100 ClinCard (reloadable debit card). All interviews were audio-recorded and transcribed for analysis.

### Analysis

The analysis of interview data was conducted in three phases. During the first phase of analysis for the parent study, two members of the research team [EC, a qualitative sociologist, and JC, a cultural anthropologist] enlisted a combination of deductive and inductive analytic techniques to code the full dataset. The research team met frequently to compare initial findings, refine themes, and develop a final codebook.

For the present study, two members of the research team [SS and EC] conducted a secondary analysis of the data to explore the experience of STS and compassion fatigue among frontline staff of post-overdose outreach programs. As part of this phase of the analysis, the two researchers examined and further refined initial codes (i.e., program origin story and personal motivations, conversations after contact with overdose survivors and social network members, procedures if fatal overdose, team training, team debrief, and processing and grief) using an iterative categorization technique [47]. The themes derived from this process were subsequently reviewed and refined by members of the broader research team to arrive at consensus.

In the final phase of the analysis, one mixed methods researcher [SF] re-read all the transcripts, further refined the codes, and organized the findings according to the three conceptual constructs (empathy, secondary traumatic stress, and compassion fatigue resilience) and related sub-areas described in Ludick and Figley’s compassion fatigue resilience framework [41], described above. This organization of results was guided and refined through discussions between multi-disciplinary members of the research team [SS, SF, JC, AY]. Findings presented here are the result of consensus between that group and the full list of authors. All qualitative data analyzed for this paper were managed within NVivo 11 software (QSR International Pty Ltd., version 11, 2017).

### Ethical review

Study procedures, including remote interview procedures necessitated by COVID-19 pandemic restrictions, were reviewed and approved by the Institutional Review Board of the Boston University Medical Center.

## Results

### Study population

Thirty-eight semi-structured interviews were conducted with staff from 11 post-overdose outreach programs in Massachusetts (Table 1). The 38 interviewees self-identified as police officers (*n* = 15), recovery coaches (*n* = 8), harm reductionists (*n* = 5), clergy members (*n* = 2), substance use disorder counselors (*n* = 2), social workers/case managers (*n* = 2), fire fighters (*n* = 1), and other outreach partners/program managers (*n* = 3). The sample was majority male (60%) and white (92%). About half (47%) reported living in the same community where they performed post-overdose outreach. Across the sample, participants reported a mean of 12 years working in their profession and an average of 2.8 years working as a post-overdose program staff member.

**Table 1:** Characteristics of interviewees, Massachusetts post-overdose outreach program staff, 2019–2020

	All Interviewees (*n* = 38)
**Professions**, n (%)	
Police Officer	15 (39.5%)
Recovery Coach	8 (21.1%)
Harm Reductionist	5 (13.2%)
Clergy	2 (5.3%)
Addiction Treatment	2 (5.3%)
Social Services/Worker	2 (5.3%)
Fire Fighter	1 (2.6%)
Other Outreach Partners/Program Managers	3 (7.9%)
**Age**, mean (SD)	41.6 (11.1)
**Race**, n (%)	
White	35 (92.1%)
Multi-Racial	2 (5.3%)
Asian	1 (2.6%)
**Ethnicity**, n (%)	
Hispanic	3 (7.9%)
**Gender Identity**, n (%)	
Female	15 (39.5%)
Male	23 (60.5%)
**Live in community where they work**, n (%)	18 (47.4%)
**Years in profession**, mean (SD)	12.0 (9.9)
**Months working/volunteering in post-overdose outreach program**, mean (SD)	33.7 (32.8)

### Summary of findings

Findings have been organized according to the three main constructs within Ludick and Figley’s [41] compassion fatigue resilience model (empathy, secondary traumatic stress, and compassion fatigue resilience) (see Table 2). Within the empathy construct, concern for others’ well-being emerged as a motivator to engage in post-overdose outreach work – with workers taking time to try to understand others’ perspectives and demonstrating cognizance of the importance protecting individuals in vulnerable situations and trust-building during outreach. Within the secondary traumatic stress construct, interviewees noted that exposure to others’ trauma through stories and disclosures was a regular and repeated occurrence – made more difficult when these exposures overlapped with workers’ personal social spheres. Within the compassion fatigue resilience construct, interviewees described the presence and absence of self-care practices and routines, social supports, and workplace supports with the potential to minimize or exacerbate the experience of STS and compassion fatigue. Job satisfaction and emotional detachment from work experiences also arose as potential protective factors. Throughout the findings, interviewees reported inconsistent presence and utilization of formal support for STS and compassion fatigue within their post-overdose outreach teams.

**Table 2:** Compassion fatigue resilience framework in the context of post-overdose outreach, Massachusetts post-overdose outreach program staff interviews, *n* = 38

**Empathy** (compassion fatigue resilience constructs and sub-constructs)	
Empathic concern	Concern for others’ well-being (theme and example quotes): “*[I was] eager to do something because I recognized the problem. I recognized people out there struggling with addiction*.”
Empathic ability	Capacity to adopt and understand others’ perspectives: “*I can empathize with the amount of stuff they’ve been through. They share a lot of trauma with us, they share a lot of what’s going on in their life*.”
Empathic response	Delivery of empathic and compassionate services: “*Just let them know it’s an honor to speak to them. It’s an honor to even be there. Thanks for opening the door is huge.*”
**Secondary Traumatic Stress**	
Trauma exposure	Exposure to others’ trauma: “*I have had situations where I went out with an officer and the person of interest actually died that morning… so that was like really difficult*.”
Trauma exposure	Trauma exposure within personal social spheres: “*So one of my latest… I knew the address, which was actually close to where I grew up.*
Trauma exposure	Prolonged/repeated exposure to trauma: “*You know, over the years… you see a lot of the ugly in society. You’re getting calls when people are suffering the most, at their worst… afraid, angry, upset, hurt.”*
**Compassion Fatigue Resilience**	
Self-care	Self-care practices and routines: “*Well, I go home and walk my dog, and exercise regularly, eat well*.”
Detachment	Detachment from outreach experiences: “*And so, you know, like anybody else when you’re dealing with things like that you get this cynical side, you get this hardened side to be self-protective*.”
Social support	Social support: “*You gotta have little breaks together, lunches [or] thing[s] to just get away from the work for a little bit.”*
Job satisfaction	Job satisfaction and occupational valuing: “*I love what I do. My experience has been great. It’s rewarding for me as an individual in recovery to work with the community*.”
Workplace support	Professional supervision and counseling support: *“I’ve sat down with [my recovery coach supervisor] and cried. It’s been like, this one [post-overdose outreach visit] just hitting home, you know, it just sucks.”*

### Empathy

#### Concern for others’ well-being (empathic concern)

Empathic concern, defined as verbally expressed compassion and interest in helping others, emerged as a unifying motivation for post-overdose outreach work across roles and professions represented in interviews with post-overdose outreach team members. Interviewees expressed genuine interest and concern for the individuals they expected to encounter when conducting post-overdose outreach. Motivations for participating in post-overdose outreach work ranged from general acknowledgement of addiction and overdose as societal issues to more personal experiences and stories. As one firefighter working on an outreach team described, “*[I] got into this work [post-overdose outreach] because, I wanted to help people.*” They went on to say, “*First and foremost, you just have to have empathy. You know, you have to care. You certainly have to care, have an interest in it, want to help*.” This sentiment was shared by a police officer, who was “*eager to do something because I recognized the problem. I recognized people out there struggling with addiction*.”

Motivations for other interviewees were more personal in nature and often included descriptions of family and friends impacted by substance use. As one police officer described, “*I initially volunteered pretty much right off the bat. I have a… a brother who’s been in recovery now for quite some time.*” A police officer with a different program, similarly noted, “*I was asked from the Deputy, actually. I kind of have a history or family history of it, so he knows before I was even hired here, I was constantly helping a family member*.” Among outreach staff participating as recovery coaches, empathic concern for others was described within the context of direct personal experience. One recovery coach said, “*Being in recovery myself, like, I had a personal, like invested interest.* This feeling was shared by a recovery coach from a different program who noted, “*It’s important to me. I’m in recovery myself. So, it’s [post-overdose outreach] something that I really identify with*.”

#### Capacity to understand and adopt others’ perspectives (empathic ability)

Empathic ability, defined as the capacity of post-overdose outreach staff to understand and adopt the position and perspective of other people, was displayed by many interviewees as they described the goals of their outreach work. Specifically, interviewees commented on what they suspected outreach recipients felt and on their perceived resource gaps and needs. A pastor working on one of the outreach teams felt many people they encountered “*don’t see their self-worth anymore*” and “*feel judged [because] people are [always] questioning their honesty, their sanity*.” A recovery coach with a different program noted, “*I can empathize with the amount of stuff they’ve been through. They [people encountered during outreach] share a lot of trauma with us, they share a lot of what’s going on in their life*.” As described by this recovery coach and other interviewees, exposure to others’ stories and disclosures related to substance use, overdose, potentially traumatic personal experiences, and general life circumstances was a regular occurrence for post-overdose outreach workers. For some outreach team members, conducting outreach was an eye-opening experience that changed their assumptions and biases about outreach recipients: “*You get to see the other side of things. You kind of get a better understanding of what someone’s life might be like, someone whose life is a lot different than yours*.” A recovery coach with a different program described trying to see beyond the substance use itself and working with people to explore and understand the “*underlying issues that feed into it*.” As one recovery coach described:

*I had more resources at my disposal when I was trying to get into recovery. And it was still so difficult to access treatment. So, it’s like I can’t imagine, like people who don’t have that. Who don’t have family. Who don’t have all of these resources at their disposal. Like, how do you navigate that?*

As illustrated by this recovery coach, some interviewees placed less emphasis on internal states, focusing on the external experiences of others.

#### Delivery of empathic and compassionate services (empathic response)

Concern for the well-being of others (empathic concern) and the capacity to adopt others’ perspectives (empathic ability) generally translated into the delivery of an empathic response during post-overdose outreach encounters. Rather than focusing on the services delivered or markers of behavioral change, many interviewees highlighted the opportunity to “*connect and hope*” or to demonstrate to overdose survivors and their families that “*the wider community cares*” and that “*somebody cares and that somebody has their best interest in mind*.” A police officer with one outreach team described their approach this way:

*I’ll sit right next to them. I’ll sit on the couch with them. I’ll go outside, sit on the porch, sit on the stairs, whatever they’re comfortable with, and just start the conversation with “What’s going on? You know, what’d you do today, you know? And… they’ll be like “Oh you know, today was tough. I had work and all that.” And I’m like “Yeah, I’ve been here for nine hours. Like I can’t wait to go home.”*

Other interviewees described attempts to forge a connection with overdose survivors and their families through the sharing of stories of lived experience. This approach was explained by a recovery coach: “*Well, a big part of it with recovery coaching is being able to tell your story. So, you know, I tell people, ‘My story is different than yours maybe, but it’s, I get it and I’m not gonna look down on you for that’.*”

For other team members, an empathic response to outreach transcended in-the-moment actions and discussion. For these interviewees it was characteristic of the overall encounter. For one harm reductionist this meant maintaining boundaries and protecting clients who are in a vulnerable situation and “*in a weak moment of their time*.” This sentiment was shared by a recovery coach who offered: “*Just let them know it’s an honor to speak to them. It’s an honor to even be there. Thanks for opening the door is huge.*” This interviewee went on to emphasize the importance of asking permission to talk and letting people know that they were under no obligation to speak, “*because some people are so intimidated by people showing up at their door that they feel they have to disclose everything*.”

### Secondary traumatic stress

#### Exposure to others’ trauma

Post-overdose outreach staff are exposed to others’ trauma, which makes them vulnerable to STS and compassion fatigue. Recounting their experiences working on post-overdose outreach teams, interviewees commonly provided descriptions of direct and indirect exposure as an aspect of their job. A recovery coach recalled, “*last week we had a client of ours pass away from a drug overdose and days like that it’s tough*.” A police officer with a different program, noted, “*We have a lot of heartache. Some of our clients do die. And it’s sad*.” A social worker remembered working with an individual who engaged with the outreach team and started accessing recovery services prior to having a fatal overdose. Discussing the impact on the team, they said, “*So, that one messed us up, and actually, we could tell a couple of the officers it hit pretty hard too*.”

While these accounts focused on learning about the death of an individual the team had already engaged with, other interviewees described unexpectedly learning about a fatal overdose event when they were conducting outreach. A harm reductionist recalled “*I have had situations where I went out with an officer and the person of interest actually died that morning… so that was like really difficult*.” A harm reductionist with another program described a situation when the team arrived to find the person being carried off in an ambulance. They remembered, “*We went to the hospital and watched them work on him for like an hour and a half, and they didn’t make it. And I remember, like, the ride back to the police station. Like, we really didn’t say anything to each other*.”

#### Trauma exposure within personal social spheres

For some interviewees, trauma exposure associated with post-overdose outreach occurred within the context of the outreach worker’s personal life and community network. This was particularly true for outreach staff working within small close-knit communities where they grew up or presently resided. One harm reductionist remembered, “*So one of my latest… I knew the address, which was actually close to where I grew up. So, I went in, the officer went with me, and the chaplain, she opened the door and then she asked me ‘Do you remember me? You know who I am?’ And I said ‘Yes.’”* A harm reductionist with a different program described a similar situation when encountering someone they went to high school with during an outreach visit. Describing the effect of these visits, they said, “*Sometimes it’s embarrassing for people just, you know, to like see me there. Or like, sometimes it’s hard because it’s emotional, you know*.” For one recovery coach, encountering social connections extended to potentially encountering individuals with whom they had formerly used substances – which they described as “*sometimes hard to separate*.”

#### Prolonged and repeated exposure to trauma

While some interviewees focused on specific trauma experiences, others emphasized prolonged and enduring exposure. As described by a pastor on one of the outreach teams, “*You know, over the years… you see a lot of the ugly in society. You’re getting calls when people are suffering the most, at their worst… afraid, angry, upset, hurt. You’re seeing it all*.” One harm reductionist described feeling like being on a roller-coaster. “*It has its up and downs, I mean, it’s people that overdose, so a lot of times [they] don’t survive… I think that’s probably the toughest part of the job*.” Other interviewees more bluntly stated that they, “*see a lot of bad things*” or “*see a lot of shit, a lot of shit, that like, is just hard to take at the moment*.” For one recovery coach, repeated exposure by some team members made the experience feel routinized. They described an experience where an outreach contact agreed to access services and the team was picking her up the next day to transport her to a facility:

*She never showed up and then she died the next day. And I remember the team, now, this is a police officer, a firefighter, and the EMT, they’re like “All right.” And I’m like “You guys don’t want to cry this out real quick, you know?” I’m a human being with feelings and this person just died. And I was like “Oh, so we’re just gonna act like nothing happened? We’re gonna go to the next call?”*

Among multi-disciplinary outreach teams like this one, each staff member has repeated exposure to trauma among the people they are trying to reach. However, staff may be in different places as far as how they respond to and cope with trauma and grief.

### Compassion fatigue resilience

#### Presence and absence of self-care practices and routines (self-care)

Self-care emerged as one of the most salient protective mechanisms utilized by post-overdose outreach staff members. Interviewees mostly described self-care strategies as a combination of individualized learned behaviors and activities they engaged in outside of the work setting, such as athletics, yoga, meditation, art, and volunteer work. One fire fighter described their personal self-care routine, “*Well, I go home and walk my dog, and exercise regularly, eat well*.” Multiple interviewees described self-care as a skill that some individuals had honed prior to joining the post-overdose outreach team based on their prior personal and professional experiences. Talking about their police and fire colleagues, one outreach specialist noted, “*They’ve seen this. They’ve already learned their coping mechanisms and know when to stop. They’re all very good. They come to this team knowing self-care.*” This perspective was echoed by a recovery coach talking about other recovery coaches on their team, “*Most are in recovery themselves. And so have done some personal growth around the idea of self-care. Defining what it is for them. In their own recovery*.” Many interviewees (mostly those who identified as recovery coaches) noted that self-care was a valued and respected practice. One recovery coach described a culture of self-care, “*We all really honor the concept of self-care. The agency itself has a, you know, environment of self-care. We really try to respect that people, you know, can only give if they have a full cup.*”

Amidst descriptions of the benefits of self-care, interviewees also noted a lack of formalized support within the outreach programs that employ them. Recovery coaches were more likely than colleagues of other professions to express this sentiment. One recovery coach described, “*This is a tough field to work in and a lot of us really struggle with self-care. We really struggle with, you know, transference, countertransference, and all that stuff.”* A recovery coach with a different program more bluntly stated, “*It is a bit of a slippery slope sometimes to reengage with the people that you’ve been trying to stay away from to maintain your own recovery. The environments, the situations, the stories, the chaotic use can all be very triggering.*”

#### Detachment from outreach experiences (detachment)

The ability to mentally disengage and distance oneself from work-related experiences is identified as a protective factor within the compassion fatigue resilience framework [41]. Multiple interviewees described how they distanced themselves from the emotive nature of certain work experiences. A pastor on one outreach team communicated this as, “*And so, you know, like anybody else when you’re dealing with things like that you get this cynical side, you get this hardened side to be self-protective*.” A police officer on the same team used the terms “*numbness*” and “shell” to capture the same idea, “*So after, you know, a while, a few years, day in, day out, you kind of get numb to it all really. Getting the numbness that… it sounds cheesy, but that shell over you, but still maintaining your humanity.*”

Multiple interviewees mentioned the use of dark humor as a detachment strategy. Dark humor was most often discussed by police and fire fighter team members or by others in reference to these occupations. One fire fighter explained, “*You tend to see humor in a lot of things that maybe the average person may not or wouldn’t…? But that’s just the way the brain copes with different things… dark humor, as they call it.*” A recovery coach also noted the use of dark humor among police officers on the outreach team, interpreting it as a type of “*stoic avoidance*” of the “*emotional impact that the work has.*”

Despite identifying multiple forms of detachment, interviewees also described the limitations of relying on detachment as a coping mechanism, especially over the long-term. As one harm reductionist described, “*you’re usually fine at that moment. But later on at night, when the shit starts to play in your head is when you’re not okay. When I wake up at four in the morning ‘cause I dreamt about it or something like that is when it hits you.*” A recovery coach in a different program similarly commented, “*There’s a culture of we’re going to suck it up and tough it out. Right? That works for a while. But it won’t work long-term. And if it doesn’t get dealt with, it’s going to come out some way, somehow*.” Other interviewees who indicated that they try not to think about it in the moment acknowledged that this was a temporary solution and that it may “*surface later in life*.” and questioned whether people can really “*automatically turn that knob off and just go back to your life*.”

#### Social support and presence of caring co-workers (social support)

Most interviewees described relying on fellow post-overdose outreach team members for social and emotional support. An outreach specialist with one program described the informal check-in process they had with their “*close team*” saying, “*You gotta have little breaks together, lunches [or] thing[s] to just get away from the work for a little bit.”* A peer support specialist from a different program also noted frequently doing “*lunches and stuff together*” with other team members. Taking breaks was seen by some as a temporary reprieve and way to shore up before returning to the job. Often, this network of support extended beyond work hours, as described by a recovery coach:

*Last week we had a client of ours pass away from a drug overdose and days like that it’s tough. But I have the pastor’s numbers. I have all the officers’ numbers where I created that relationship where I call them and just talk to them. And for me, my support network is the people that I work with…if they’re feeling down and out and they need someone to talk to because of this job my door is always open for them too.*

The tactic of relying on other team members appeared contingent on team cohesion and closeness: if teams felt close, mutual reliance was a viable option. When teams were not cohesive, isolated self-processing appeared more likely to occur. One recovery coach captured this dynamic by saying. “*It’s a matter of having great relationships with our coworkers and being able to trust that it’s okay for me to sit there and sob if I have to*.”

Shared time together in the car traveling to and from outreach visits emerged as a safe and opportunistic setting for team members to debrief visits and provide and receive support from co-workers. As described by a recovery coach, “*We get back into the car and we just regroup our thoughts. So, we can all let out our emotions because after an intervention it can be draining sometimes*.” Other interviewees described valuing “*opportunities to talk*” on car rides and “*getting to know*” other team members. For outreach team members who primarily work in different agencies than their outreach partners, the vehicle became their shared office space or shared work environment.

#### Job satisfaction and occupational valuing (job satisfaction)

Almost all interviewees expressed a general sense of satisfaction with their work and used terms such as “*rewarding*” and “*positive*” to describe its impact on them. Occupational valuing, the ways individuals attribute meaning and importance to their work, varied with expectations of the outcomes of outreach. For some interviewees, the value attributed to outreach was “*getting [people] to a better place and watching them grow unto a whole different person*” or the feeling that the outreach worker has “*some power to make a positive impact.*” Other interviewees commented more broadly on just “*being present*” and “*making a connection*” with individuals who otherwise would not be reached with offers of assistance. Recovery coaches were likely to frame job satisfaction and occupational valuing in reference to their own past experiences. As described by one recovery coach, “*I am also in recovery myself, so being active and out here has helped me a lot, personally. So, it’s been very, very positive in my life to be part of it now, doing the opposite*.” Another recovery coach similarly commented, “*I love what I do. My experience has been great. It’s rewarding for me as an individual in recovery to work with the community*.” Other interviewees drew value from all being committed to a coordinated “*common cause*” and shared mission.

#### Professional supervision and counseling support (workplace supports)

There was a notable absence in descriptions of the presence of workplace support activities among interviewees, with available support often determined by profession. Several interviewees indicated that their programs *“probably don’t do enough”* to specifically support staff and that formalized programming was *“something we should look into more.”*

A minority of interviewees pointed to either specific or general mechanisms in place that supported staff. For example, a harm reductionist described how the post-overdose outreach team had recently completed a retreat, “*we just had one I think last month or two months ago where we shut down the shop, we went to a Zen Center*;” however, this kind of specificity was uncommon among interviewees. A post-overdose outreach specialist and licensed mental health counselor described how post-overdose outreach team members were already equipped to manage issues encountered in post-overdose outreach work and therefore the outreach team did not need any team-specific support activities. *“Each one of our firefighters and police officers have all been 10 years plus. They’ve seen this. They’ve already learned their coping mechanisms and know when to stop.”*

Some interviewees described one-on-one discussions with their supervisor as a place to unpack experiences and discuss the emotional impacts of post-overdose outreach visits, as was the case for one recovery coach. *“I’ve sat down with [my recovery coach supervisor] and cried. It’s been like, this one [post-overdose outreach visit] just hitting home, you know, it just sucks.”* According to this interviewee, individuals had to have the desire to seek out support. While not all interviewees shared a desire to receive institutional support, all post-overdose outreach team members who had been involved in such activities endorsed its value.

Interviewees described varying levels of availability and utilization of group and individual supervision and counseling support services. When support services for outreach team members were available, it was common for this programming to be optional, with the onus placed on team members to advocate for what they needed and to seek it out themselves. A police officer described Tuesday night meetings with recovery coaches and grief specialists who provided ongoing training and support for the post-overdose outreach team. *“We just go around the room and we just talk about different things… they [post-overdose outreach team members] just learn so much from these people.”* Due to lack of attendance, however, these meetings were discontinued. A post-overdose outreach specialist described the dynamic on their team where first responder colleagues (police, fire, and EMT) chose not to attend support initiatives. *“We’ve been offered group supervision; we’ve been offered grief counseling; we’ve been offered to have somebody come in and talk to. They don’t want it. I can’t make ‘em do it.”* As this interviewee noted, professional culture sometimes affected whether team members sought support or acknowledged the need for it. Most interviewees also recognized differences in the types of support that team members across professions might need or be able to access.

A minority of interviewees shared that they did not experience work-related stress while working in post-overdose outreach. When asked if their post-overdose outreach team did anything to cope with potential grief, one police officer responded, *“I don’t think we’ve experienced anything that would make you feel like you need to do something like that. ‘Cause usually the traumatic stuff is the overdose itself… It’s usually a more positive situation with the follow-ups.”* Several law enforcement interviewees, including this interviewee, described their engagement with post-overdose outreach as a distinct and positive counterpoint to the other work they did within their professional duties and less intense than the other situations they may encounter on the job. For these interviewees, support was not considered necessary, because post-overdose outreach events were not perceived as prompting secondary traumatic stress.

## Discussion

In this qualitative study, we explored elements of professional post overdose outreach work that might contribute to or mitigate the experience of STS and compassion fatigue among post-overdose outreach team members. Specifically, we assessed whether upstream factors were in place (such as empathy and repeated exposure to trauma) that may contribute to the occurrence of STS and compassion fatigue. We then examined the presence of compassion fatigue resilience risk and protective factors placing post-overdose outreach workers at higher or lower levels of risk over time. Post-overdose outreach program team members described the compassion, perspective-taking, and empathic approach they adopted during post-overdose outreach. They discussed the repeated exposure to trauma faced through participation in outreach work. Lastly, post-overdose outreach workers outlined a variety of strategies used in the context of outreach work, including self-care practices, social support offered by co-workers and professional supervision, occupational valuing, and practicing detachment from experiences.

Interviewees in our study described post-overdose outreach work as both rewarding and challenging. On one hand, they found purpose in work they personally believed was making a difference in their communities, and yet, interviewees almost universally reported that this kind of engagement took an emotional toll. Previous research has highlighted the need to provide time and space within organizations for such multifaced emotional management strategies, including the dark humor that interviewees in our study described [37, 48]. Mamdani et al. [30] found that finding meaning in one’s work is a key motivator for engagement in overdose response, suggesting that post-overdose outreach workers may continue in their work, despite the associated stress and loss. Yule and Levin [49] specifically recommend strengthening training in post-intervention needs, developing written protocols for providers, and taking concrete steps beyond the organization, such as attending the funerals of patients who pass from overdose as a way to collectively grieve.

Compassion fatigue is characterized by a physical, emotional, and spiritual depletion in the capacity to care among those on the frontlines of caregiving for traumatized groups [19, 24, 50–52]. Compassion fatigue can affect job satisfaction, reduce productivity, and negatively impact the delivery of healthcare services [9, 53].﻿ Many post-overdose outreach team members interviewed for this study reported working in post-overdose outreach for multiple years, with consistent exposure to stressful events, in support of Ludick and Figley’s repeated exposure construct of the compassion fatigue resilience model [41]. There are layered concerns about compassion fatigue for post-overdose outreach workers that warrant attention. Unattended and unsupported traumatic stress can contribute to personal and professional burnout among outreach team members. This could, in turn, negatively impact the care provided to overdose survivors and their social networks – including the re-traumatization of overdose survivors and their social networks, decreased levels of engagement, poor quality interactions during outreach, and ultimately, worse health outcomes for overdose survivors.

Strikingly, most interviewees stressed the lack of formalized support systems within their place of work and the resilience strategies they employed in the absence of support for undeniably challenging work. Both proactive and responsive interventions to prevent compassion fatigue are warranted for post-overdose outreach staff and persons who work within these programs. Cook [54] recommends, in addition to relying on individual, prescriptive interventions, that agencies and agency leadership focus attention on organizational culture shifts and formalize infrastructure to provide support. Practically, organizations may consider providing opt-out supervision and acute care support (automatic enrollment in support activities), including fixed and proactive debriefing sessions that incorporate expert clinical supervision. Making staff support such as helping post-overdose staff develop compassion fatigue resilience strategies an organizational objective and a cornerstone of post-overdose outreach program implementation could better decrease susceptibility to STS and compassion fatigue. Recently published best practice guidance for post-overdose outreach programs identified the need for all team members to receive training, support, and supervision to mitigate the negative effects of direct and secondary trauma exposure [55]. Post-overdose outreach teams bring together a diverse array of professionals who approach substance use and experience STS and compassion fatigue in disparate ways that are often informed by the other professional environments in which they are embedded. Compassion fatigue resilience structures should be customized to the professional cultures, geography, and lived experience of post-overdose outreach team members to increase acceptability and utilization. Post-overdose outreach programs in smaller or tight-knit communities may need specialized support for instances in which overdose survivors and decedents are personally known to post-overdose outreach team members. Beyond this, post-overdose outreach programs may need to seriously consider the degree to which their own program models and modes of service delivery are artificially inflating the personal, interpersonal, and secondary stress experienced by outreach workers. As one interviewee is quoted above, “*[outreach recipients] are so intimidated by [outreach team members] showing up at their door that they feel they have to disclose everything.*” Setting aside the question of whether it is ethically or therapeutically preferable to contact and gain consent from outreach recipients prior to in-person visits or to attempt an outreach visit unannounced (both of which were reported as standard procedures by different programs participating in the parent study), the data presented here offers reason to question whether outreach programs operate in line with best practices already established by skilled professionals in the realms of social work, family services, assertive treatment, and other domains where community-based interactions are common. Additionally, previous findings from our research group indicate that while Massachusetts municipalities with high numbers of opioid-related emergency responses who implemented post-overdose outreach programs experienced a statistically significant lower rate of opioid fatality rates over time compared to municipalities that did not implement such programs, the elements of post-overdose outreach expected to shape outcomes, including naloxone distribution and intensity of outreach, did not impact community-level overdose rates in the municipalities where these programs were implemented [7]. Taken with the findings of this analysis, that post-overdose outreach work is stressful and often unsupported, future research is necessary to determine what ingredients of post-overdose outreach may benefit overdose survivors and social networks and in what contexts. A worst-case scenario, in this regard, might constitute a confluence of ill-advised practices that result in greater-than-necessary stress for outreach team members, lower quality outreach services, worse service outcomes for overdose survivors as a result of that stress, and increased risk of secondary trauma or re-traumatization of outreach recipients. Whether certain program models unintentionally increase these risks for staff and survivors has not been evaluated and, based on the data presented here, some but not all outreach team members are even considering these possibilities.

Special consideration may also be warranted for team members with lived experience of substance use or with substance use disorder – which may add a personal and/or compounding layer of complexity to experiences of overdose grief. Mamdani et al. [30] conducted focus groups with Canadian peer workers, individuals who were hired by community organizations because of their past or present drug use experience, who were involved in overdose response initiatives. Peer workers in that study described the variety of stressors they experienced in this work, including the constant exposure to trauma. Programs should consider whether and what additional or customized STS and compassion fatigue supports might be necessary to cope, first, with the needs of each team member and, second, with the needs of the team as a whole—versus uncritically adopting a uniform approach to support.

Future research should assess the time team members spend in transit to and from post-overdose outreach visits together as opportunities for team processing, including team preparation, support, and debriefing, as these venues were mentioned by several interviewees as times of post-overdose outreach planning and reflection. Specific time set aside for team processing warrants feasibility testing and evaluation in post-overdose outreach settings, such as the low threshold “Hero Help” intervention aimed at attenuating effects of emotional burnout among police officers by highlighting the value of post-overdose outreach work through the sharing of success stories [56]. At the individual level, research among community health workers has identified mindfulness activities, like meditation and guided imagery, to be effective at reducing burnout among staff, with interventions focused on improving compassion fatigue resilience appearing most effective [21]. Previous work in Massachusetts suggests that service providers may benefit most when grief coping skills and support are integrated into robust staff training programs that also focus on coping with trauma and distress in the context of the overdose response environment [54]. This work advises that professionals may benefit most when support is included in the aims of organizational policy and tailored to local program staff needs and concerns.

The study presented here has limitations. Qualitative data collection occurred in Massachusetts in 2019 and 2020 and thus may not be fully generalizable to other geographic regions. Additionally, while STS and compassion fatigue support activities may not be available through post-overdose outreach programs, activities that do exist may lack advertisement, or the structures that do exist are not experienced by participants as responding to elements of their distress. This study cannot address these questions. Lastly, our sample was predominantly white and majority male. Post-overdose outreach staff of other races and/or gender identities may process STS and compassion fatigue differently. The intersection of racism and STS, which this study did not explore, is especially important to understand in the context of post-overdose work. The views and experiences of post-overdose outreach staff representing a wider array of social and racialized identities, who were not sufficiently captured by this study, should be the focus of future research.

## Conclusion

As post-overdose outreach programs proliferate, post-overdose outreach staff will regularly face experiences that may contribute to secondary traumatic stress and compassion fatigue. Emotional burdens on post-overdose workers are high and enduring. Formal strategies for resilience are few, often private, and opt-in. Dedicated time for team processing of overdose deaths and other stressful encounters should be considered to create robust institutional resilience supports for the secondary traumatic stress and compassion fatigue experienced in the context of this work.

## Data Availability

The datasets generated and/or analyzed during the current study are not publicly available due to the qualitative nature of the data analyzed and thus the potential for individual privacy to be compromised.
